# Particulate Matter Decreases Intestinal Barrier-Associated Proteins Levels in 3D Human Intestinal Model

**DOI:** 10.3390/ijerph17093234

**Published:** 2020-05-06

**Authors:** Brittany Woodby, Maria Lucia Schiavone, Erika Pambianchi, Angela Mastaloudis, Shelly N. Hester, Steven M. Wood, Alessandra Pecorelli, Giuseppe Valacchi

**Affiliations:** 1 Plants for Human Health Institute, Animal Sciences Dept., NC Research Campus Kannapolis, State University, Kannapolis, NC 28081 USA; blwoodby@ncsu.edu (B.W.); schiavone3@student.unisi.it (M.L.S); epambia@ncsu.edu (E.P.); apecore@ncsu.edu (A.P.); 2 Pharmanex Research, NSE Products, Inc., Provo, UT 84601, USA; mastaloudis@gmail.com (A.M.); shester@nuskin.com (S.N.H.); stevew@nuskin.com (S.M.W.); 3 Department of Biomedical and Specialist Surgical Sciences, University of Ferrara, 44121 Ferrara, Italy

**Keywords:** pollution, 4HNE, ZO−1, pollution, tight junctions, desmosome

## Abstract

(1) Background: The gastrointestinal tract (GI) tract is one of the main organs exposed to particulate matter (PM) directly through ingestion of contaminated food or indirectly through inhalation. Previous studies have investigated the effects of chronic PM exposure on intestinal epithelia in vitro using Caco−2 cells and in vivo using mice. In this study, we hypothesized that chronic PM exposure would increase epithelial permeability and decrease barrier function due to altered redox homeostasis, which alters levels and/or localization of barrier-associated proteins in human three-dimensional (3D) intestinal tissues. (2) Methods: Transepithelial electrical resistance (TEER) in tissues exposed to 50, 100, 150, 250, and 500 µg/cm^2^ of PM for 1 week and 2 weeks was analyzed. Levels and localization of tight junction proteins zonula occludens protein 1 (ZO−1) and claudin−1 and desmosome-associated desmocollin were analyzed using immunofluorescence. As a marker of oxidative stress, levels of 4-hydroxy-nonenal (4HNE) adducts were measured. (3) Results: No differences in TEER measurements were observed between exposed and un-exposed tissues. However, increased levels of 4HNE adducts in exposed tissues were observed. Additionally, decreased levels of ZO−1, claudin−1, and desmocollin were demonstrated. (4) Conclusion: These data suggest that chronic PM exposure results in an increase of oxidative stress; modified levels of barrier-associated proteins could possibly link to GI tract inflammatory conditions.

## 1. Introduction

Considering that 9 million premature deaths occur every year due to exposure to air pollution [1,2], the effects of pollution on human health, which range from respiratory and cardiovascular dysfunction to skin aging and gastrointestinal (GI) stress [3,4,5,6], are of great public health interest. One of the most dangerous anthropogenic pollutants is particulate matter (PM), generated directly by emissions from oil refineries, cars, aircrafts, and ships or secondarily by cross reactions with other components [7,8]. 

PM itself is a heterogenous, complex mixture of airborne solid, liquid, or mixed-phase particles that is composed of inorganic, organic, and organometallic compounds that are suspended in the air, such as polycyclic aromatic hydrocarbons (PAHs), endotoxin, and transition metals (iron, copper, nickel, zinc, and vanadium) [9]. Although the composition of PM varies between different environments, exposure has been correlated with increased morbidity and mortality [3,10], not only in people with an increased susceptibility to PM-related health conditions, including children and elderly people, but also in the healthy adult population [11]. Thus, PM exposure is a major health concern for the global population. 

PM exposure can negatively affect the physiology of a variety of organs, including the heart, lungs, skin, and the gut [3,4,5,6]. In fact, exposure of the GI tract to PM is associated with a variety of gut conditions, including inflammatory bowel disease, Crohn’s disease [12,13,14], and colorectal cancer [15,16]. The GI tract can be directly exposed to PM via ingestion of contaminated food, since 10^12^−10^14^ particles are ingested daily by an individual [13,14]. In addition, the GI tract can be indirectly exposed to PM when inhaled particles are taken up by lung-associated macrophages, transported to the luminal side of the epithelium, and carried to the oropharynx, where they can then enter the GI tract through the act of swallowing [17,18]. The primary mechanism by which PM exposure results in the development/exacerbation of GI tract disorders is believed to be due to altered redox homeostasis, as shown in other organs, resulting in a “leaky gut” condition and the secretion of gut-associated bacterial metabolites and proinflammatory mediators.

The structure of the gut is similar across all the layers of the GI tract, including both the upper (mouth, esophagus, stomach) and lower (small intestine and large intestine) sections, composed of a layer of columnar epithelial cells supported by connective tissue and smooth muscle. There are various types of cells in the gut, including goblet cells (mucus producing cells), enteroendocrine cells (hormone producing cells), and enterocytes. To prevent leakage of intestinal contents into the bloodstream, these cells are held together by tight junctions near the apical surface and desmosomes. Tight junctions are composed of multiple proteins located at the apical ends of adjacent intestinal epithelial cells [19,20]. In these complexes, integral transmembrane proteins, including claudins, occludin, and junctional adhesion molecules interact intracellularly with cytosolic scaffold proteins, such as zonula occludens proteins, which anchor these transmembrane proteins to the actin cytoskeleton [19,20]. In addition to tight junctions, the barrier function of the intestinal epithelium is also established by desmosomes. These junctions are located on the lateral side of plasma membranes and are composed of cadherin family members, including desmocollin, that can interact with each other via their extracellular domains and are associated with the cytoskeleton through interactions with specific desmosomal plaque proteins [21,22,23,24]. Interestingly, redox status has been shown to regulate the levels and localization of proteins involved in intestinal barrier function [25,26,27]. 

PM exposure has been shown to alter redox homeostasis and intestinal permeability in Caco−2 cells and in mice, but, considering the limitations of these models, we wanted to examine whether PM exposure altered the levels and/or localization of intestinal barrier-associated proteins in three-dimensional (3D) human intestinal models [28,29]. Indeed, Caco−2 cells have been shown to have altered production of cytokines and altered expression of receptors and transporters, compared to native intestinal models [30,31,32]. In addition, this model does not incorporate the use of stromal cells, which produce extracellular matrix (ECM) that is important in cellular crosstalk and in regulating levels of barrier proteins [33]. Furthermore, studies on the effects of PM exposure on the gut in vivo using mice are limited by the fact that the intestinal structure of mice differs from that of humans (i.e., longer villi and lack of mucosal folds, etc.) [34]. However, 3D intestinal epithelial models, which incorporate stromal cells and ECM, are becoming increasingly useful models due to the similarity between the morphology and behaviour of cells in these models and cells in vivo [32]. In fact, the model used in this study closely resembles native human small intestinal tissue and forms brush borders, tight junctions, and secretory granules [35]. Therefore, the purpose of this study was to analyze the effects of PM exposure on intestinal permeability and associated barrier proteins in 3D models, since these tissues not only have epithelial cells but also other cells that are important for crosstalk. In this study, we hypothesized that chronic PM exposure would increase epithelial permeability and decrease barrier function due to altered redox homeostasis, which alters levels and/or localization of barrier-associated proteins in human three-dimensional (3D) intestinal tissues. We did not observe differences in transepithelial electrical resistance (TEER) between exposed and un-exposed tissues, regardless of length of exposure, likely due to the complexity of our model. However, in support of previous work [28,36,37], we observed increased levels of the oxidative stress marker 4-hydroxynonenal (4HNE) adducts in tissues exposed to PM. Since redox homeostasis is also linked to barrier function, we examined levels of tight junction proteins zonula occludens protein 1 (ZO−1) and claudin−1 and observed decreased levels of these proteins in tissues exposed to PM for 1 week and 2 weeks. Furthermore, we observed decreased levels of desmosome-associated protein desmocollin in tissues exposed to PM for 1 week and 2 weeks. In conclusion, we have shown that chronic PM exposure alters levels of proteins involved in intestinal barrier function in 3D human intestinal tissues, potentially contributing to a leaky gut and the development/exacerbation of gastrointestinal disorders.

## 2. Materials and Methods

### 2.1. Tissue Culture

To examine the effects of particulate matter on human 3D intestinal tissues, we purchased 3D EpiIntestinal tissues from MatTek Life Sciences (MA, USA). In this model, small intestinal epithelial cells are harvested from post-mortem donors (with IRB approval), seeded onto cell culture inserts, raised to the air-liquid interface, and cultured in specially formulated medium to induce differentiation for 2 weeks [35]. This model closely resembles human small intestinal tissue and forms brush borders, tight junctions, and secretory granules [35]. On the day of arrival, inserts containing 3D EpiIntestinal tissues (MatTek Life Sciences, MA, USA) were transferred from agarose packaging onto hanging-top lids under sterile conditions. One hundred microlitres of serum-free Small Intestinal Tissue Model (SMI-100) media (MatTek Life Sciences, MA, USA) were pipetted onto the apical surface of each tissue. Subsequently, the tissues were placed in a humidified incubator overnight at 37 °C in 95% air/5% CO_2_, and experiments were performed over the course of two weeks.

For experiments, a stock solution of 4.3 mg/mL was prepared from urban particulate matter standard reference material (SRM) 1648a, which is a certified collection of atmospheric particulate matter (National Institute of Standards and Technology) that was prepared from urban particulate matter collected over 12 months from 1976 to 1977 in the St. Louis, MO area in the U.S. This stock solution was diluted into solutions with final concentrations of 500, 250, 150, 100, and 50 µg/cm^3^ in media after sonication for 30 min to prevent particle aggregation. The solutions with final concentrations of 50, 100, 150, 250, and 500 µg/cm^2^ were then vortexed prior to application to the tissues. 3D EpiIntestinal tissues were treated with the various concentrations of SR1648a (50, 100, 150, 250, and 500 µg/cm^2^) every day for 1 week or 2 weeks or with vehicle control (media alone). 

### 2.2. Transepithelial Resistance (TEER) Measurements

After 24 h of exposure to the various concentrations of particles, transepithelial electrical resistance (TEER) of 3D human intestinal tissues was assessed using the Millicel ERS2 Resistance System, as previously assessed in Caco−2 cells by Mutlu et al. 2011 [28]. Plates were equilibrated at room temperature (RT) for 20 min. Only recorded measurements that were maintained for 10 sec (signifying stability) were recorded. Blank measurements were assessed using an empty insert. Only tissues with TEER values greater than 250 Ω × cm^2^ (after subtraction from empty insert) were used, as previously described [28]. Measurements were taken daily.

### 2.3. Tissue Collection 

After 1 week or 2 weeks of experiments, 3D intestinal tissues were collected in histology cassettes and kept in 10% neutral-buffered formalin for 24 h. Then, the tissues were dehydrated in increasing concentrations of alcohol (70% to 100% ethanol) and cleared in xylene. After immersion in xylene, sections were embedded in paraffin for stability.

### 2.4. Tissue Immunofluorescence 

Paraffin sections of 3D human intestinal tissues (4 µm) were deparaffinized in xylene and rehydrated in decreasing concentrations of alcohol (100 − 30%) to water. Antigen retrieval was performed using heat-based epitope retrieval with 10 mM sodium citrate buffer (AP9003−500, Thermo Fisher Scientific, USA) (pH 6.0) at a sub-boiling temperature in pressure cooker (Instant Pot Duo using the steam setting) for 15 min. After cooling for 30 min, sections were washed 2 times for 5 min in PBS, blocked with 2% BSA in PBS at RT for 45 min, and incubated overnight at 4 °C with primary antibodies for Desmocollin (Santa Cruz sc−388590) diluted 1:50 in 2% BSA in PBS, ZO−1 (Cell signaling #13663) diluted 1:400 in 2% BSA in PBS, 4HNE (AB5605 Millipore Corp., USA) diluted 1:400 in 2% BSA in PBS, and Claudin−1 (Santa Cruz sc−166338) diluted 1:50 in 2% BSA in PBS. After overnight incubation, the sections were washed 3 times in PBS for 5 min, followed by a 1 h incubation with fluorochrome-conjugated secondary antibodies (A11004 Alexa Fluor 568, A11008 Alexa Fluor 488, and A11055 Alexa Fluor 488) at 1:1000 dilutions in 2% BSA in PBS at RT for 1 h, and then washed with PBS 3 times for 5 min. Nuclei were stained with DAPI (D1306, Invitrogen) for 2 min in PBS at RT, and sections were then washed with PBS and then water. The sections were mounted onto glass slides using PermaFluor mounting media (ThermoFisher Scientific) and imaged via epifluorescence on a Zeiss LSM10 microscope equipped at 63× magnification. Images were quantified using ImageJ [38].

## 3. Results

### 3.1. Effect of Particulate Matter On Transepithelial Electrical Resistance (TEER) in 3D Tissues

**Figure 1:**
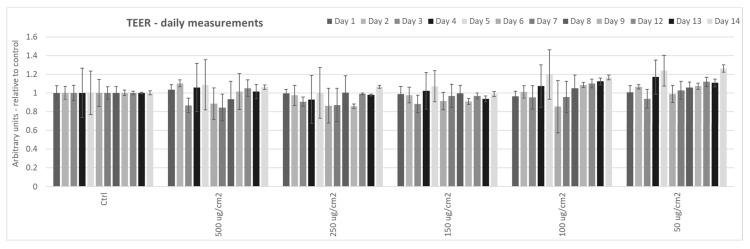
Effects of particulate matter exposure on the transepithelial electrical resistance (TEER) of 3D human intestinal tissues. TEER was analyzed in 3D human intestinal tissues that were exposed to particulate matter after 24 h at the following doses: 50, 100, 150, 250, and 500 µg/cm^2^ daily for two weeks. Data are expressed as arbitrary units (averages ± SEM of two experiments, * *p* < 0.05).

Since other labs have previously demonstrated that exposure of intestinal epithelial cells (Caco−2) to PM caused increased epithelial permeability, as measured by TEER, in cellular monolayers [28,29], we wanted to determine whether exposure of 3D human intestinal tissues to PM altered permeability. We analyzed TEER in tissues exposed to various concentrations of PM (50, 100, 150, 250, and 500 µg/cm^2^) every day for 2 weeks. However, we did not observe any differences in TEER in tissues exposed to PM at any of the assessed doses, compared to un-exposed tissues (Figure 1). We believe that these results are likely due to the fact that these tissues incorporate various types of cells, including enterocytes, M cells, tuft cells, intestinal stem cells, and paneth cells into intestinal villi [35].

### 3.2. PM Exposure Increased 4-Hydroxynonenal Levels in 3D Human Intestinal Tissues 

**Figure 2:**
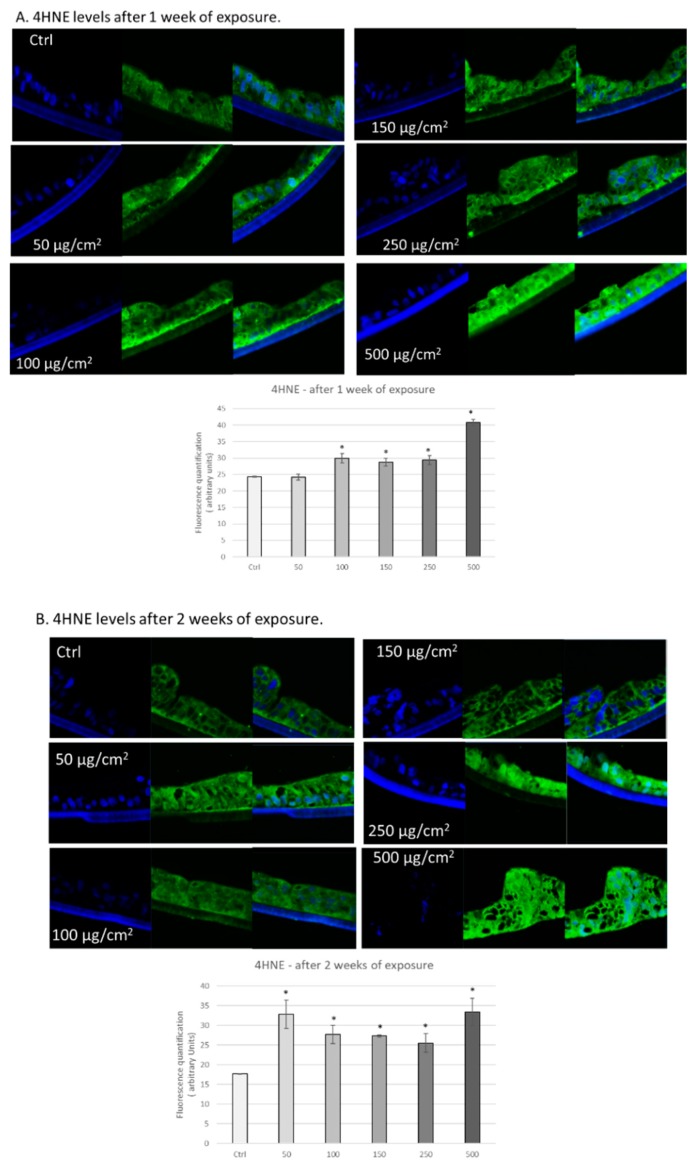
Exposure to particulate matter increased levels of 4HNE after 1 week and 2 weeks of exposure. Representative immunofluorescence images of levels of 4HNE (green color) in human 3D intestinal tissues after 1 week (**A**) or 2 weeks (**B**) of exposure with the following doses of PM: 50, 100, 150, 250, and 500 µg/cm^2^. Green staining represents 4HNE. Blue staining represents DAPI. Quantification of at least 3 independent images taken of stained tissues from two independent experiments is represented in graphs A and B. Data are expressed as arbitrary units (averages ± SEM of two experiments, * *p* < 0.05).

We hypothesized that exposure to PM would result in increased reactive oxygen species (ROS), as previously observed by other labs [28,36], due to the presence of transition metals in the particles (produce ROS via Fenton or Fenton-like chemistry) and production of redox-active quinones [37]. As a measure of oxidative damage, we assessed levels of 4-hydroxynonenal (4HNE) protein-adducts. 4HNE is a lipid peroxidation product that is highly reactive and can form protein adducts, resulting in mitochondrial damage and DNA damage [39]. As shown in Figure 2A, we observed increased levels of 4HNE adducts in 3D intestinal tissues exposed to different doses of PM for one week, ranging from 100 to 500 µg/cm^2^. Similarly, we also observed increased levels of 4HNE adducts in 3D intestinal tissues exposed to all the various concentrations of PM for two weeks (50, 100, 150, 250, and 500 µg/cm^2^) (Figure 2B). These data suggest that chronic exposure to PM induces oxidative stress in human 3D intestinal tissue.

### 3.3. PM Exposure of 3D Human Intestinal Tissues Decreases Levels of Tight Junction Proteins and Desmocollin

**Figure 3:**
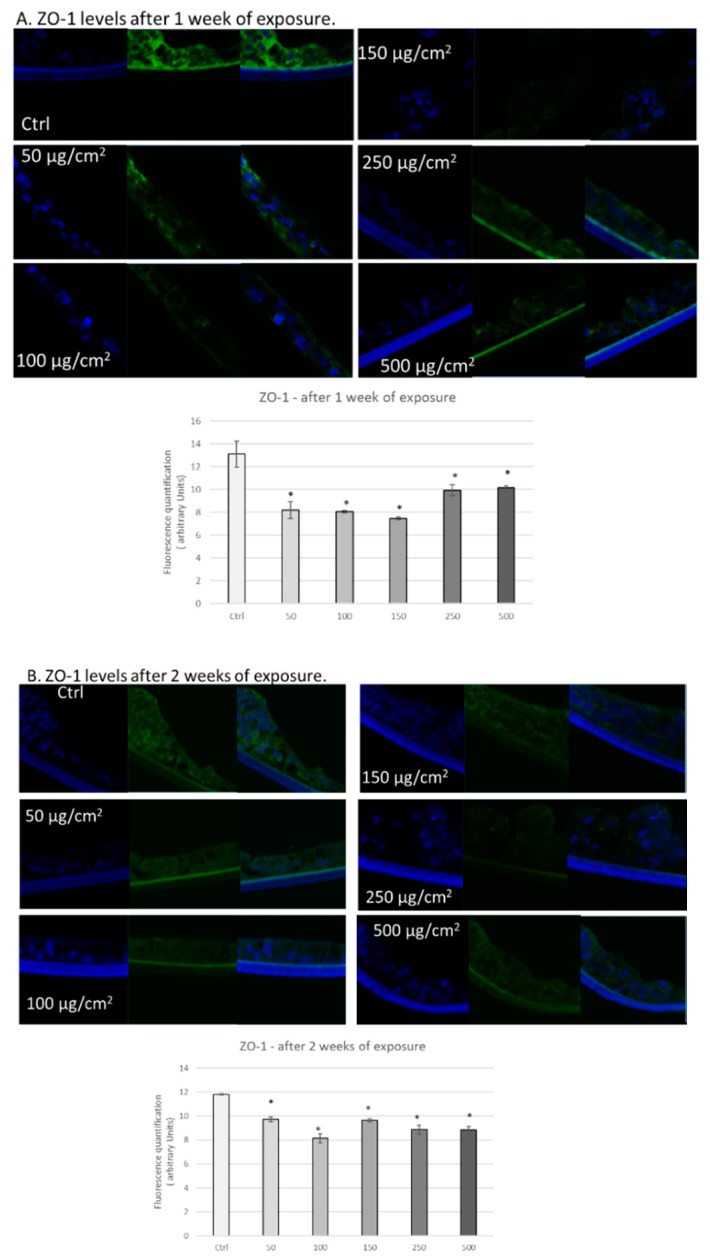
Exposure to particulate matter decreased levels of ZO−1 after 1 week and 2 weeks of exposure. Representative immunofluorescence images of levels of ZO−1 (green color) in 3D intestinal tissues after one week (**A**) or two weeks (**B**) of exposure with the following doses of PM: 50, 100, 150, 250, and 500 µg/cm^2^. Green staining represents ZO-1. Blue staining represents DAPI. Quantification of at least three independent images taken of stained tissues from two independent experiments is represented in graphs A and B. Data are expressed as arbitrary units (averages ± SEM of two experiments, * *p* < 0.05).

Next, we wanted to determine whether exposure to PM altered levels and/or localization of tight junction proteins, since redox status has been previously shown to regulate levels and localization of these proteins [25,26,27]. First, we analysed the effects of PM exposure on the levels of zonula occludens protein 1 (ZO−1). This protein is a peripheral membrane protein that stabilizes tight junctions, connecting transmembrane proteins like occludin to the cytoskeleton [40,41,42], and loss of this protein is correlated to tight junction dysregulation [43,44]. As depicted in Figure 3A, we observed decreased levels of ZO−1 in tissues exposed to all of the various concentrations of PM (50, 100, 150, 250, and 500 µg/cm^2^) after 1 week (Figure 3A). After two weeks of exposure, we observed a similar trend (Figure 3B). 

**Figure 4:**
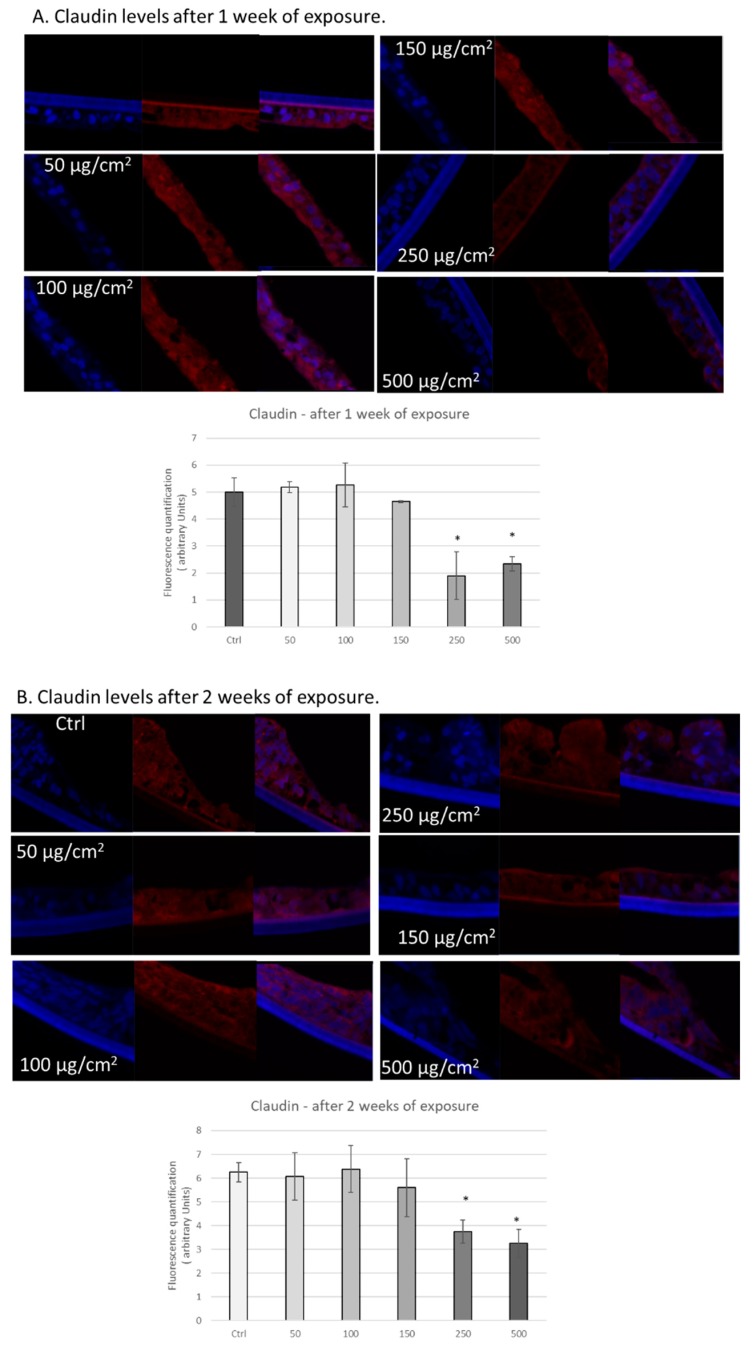
Exposure to particulate matter decreased levels of claudin after one week and two weeks of exposure. Representative immunofluorescence images of levels of claudin (red color) in EpiIntestinal tissues after one week (**A**) or two weeks (**B**) of exposure with the following doses of PM: 50, 100, 150, 250, and 500 µg/cm^2^. Red staining represents claudin. Blue staining represents DAPI. Quantification of at least three independent images taken of stained tissues from two independent experiments is represented in graphs A and B. Data are expressed as arbitrary units (averages ± SEM of two experiments, * *p* < 0.05).

In addition to ZO−1, we also analysed the effects of PM exposure on the tight junction protein claudin−1. This protein is a major component of tight junction complexes and regulates epithelial permeability via interaction with other claudin family members [45,46,47]. After 1 week of exposure, we observed significant decreases in claudin−1 in tissues exposed to 250 and 500 µg/cm^2^ of PM (Figure 4A). We observed a similar trend after two weeks of exposure with significant decreases of claudin−1 in tissues exposed to 250 and 500 µg/cm^2^ of PM (Figure 4B).

**Figure 5:**
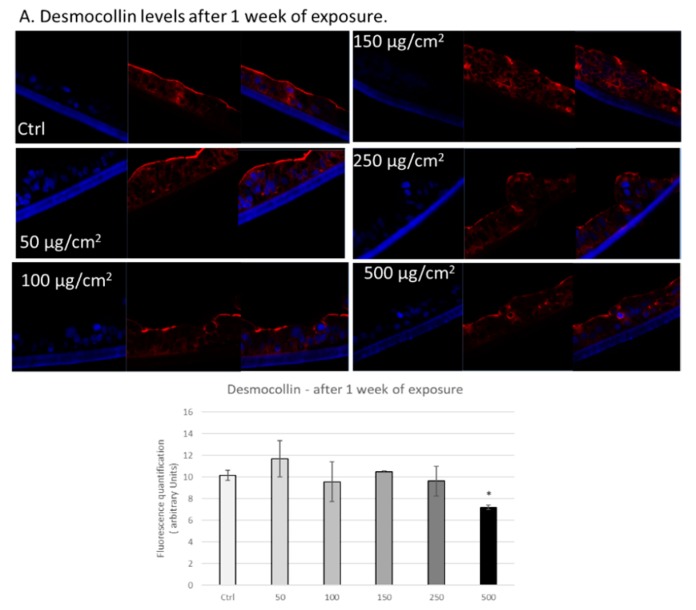

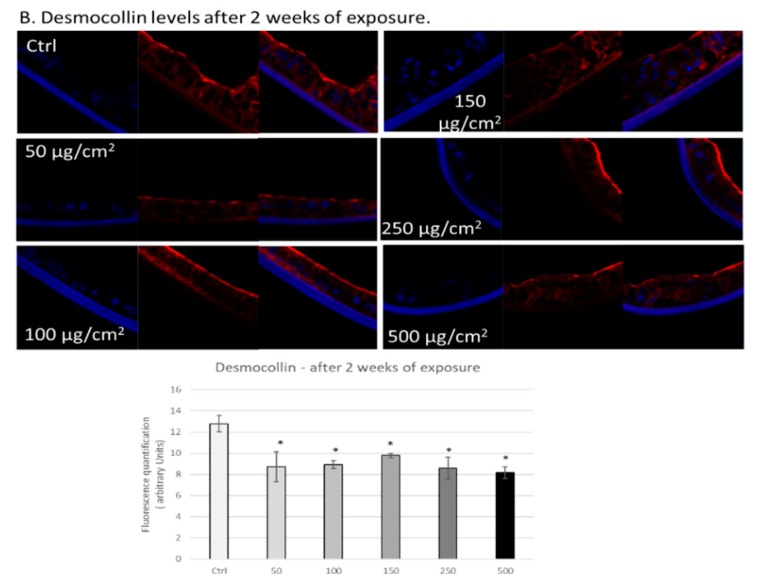
Exposure to particulate matter decreased levels of desmocollin after one week and two weeks of exposure. Representative immunofluorescence images of levels of desmocollin (red color) in 3D intestinal tissues after one week (**A**) or two weeks (**B**) of exposure with the following doses of PM: 50, 100, 150, 250, and 500 µg/cm^2^. Red staining represents desmocollin. Blue staining represents DAPI. Quantification of at least three independent images taken of stained tissues from two independent experiments is represented in graphs A and B. Data are expressed as arbitrary units (averages ± SEM of two experiments, * *p* < 0.05).

In addition to tight junction proteins, we also analysed the effects of PM exposure on the levels of the desmosome protein desmocollin in 3D intestinal tissues. This family of proteins mediates cell-cell adhesion through intracellular interactions with cytoskeletal proteins and intercellular interactions with proteins on adjacent cells [21,22,23,24]. After 1 week of exposure, we observed significantly decreased levels of desmocollin in tissues exposed to 500 µg/cm^2^ of PM (Figure 5A). After two weeks of exposure, we observed significant decreases in desmocollin levels in tissues exposed to all of the various doses of PM (50, 100, 150, 250, and 500 µg/cm^2^) (Figure 5B). Furthermore, we observed alterations in the localization of desmocollin, primarily detecting positive staining on the upper portion of the epithelium in tissues exposed to 250 and 500 µg/cm^2^ of PM after two weeks (Figure 5B).

## 4. Discussion

As a consequence of the deleterious effects on a variety of organs, research in the toxicological field is focusing on the effects of pollution exposure on specific organs. In particular, scientific interest in the effects of pollutant exposure on the GI tract is increasing, due to the fact that 10^12^−10^14^ particles are ingested daily by an individual [13,14]. Furthermore, it became apparent during the 2008 Olympic games that excessive levels of air pollution (including PM) contributed to impairments in foreign athlete performance, GI distress, and systemic inflammation [48].

However, only a few studies have investigated the effects of pollution on the GI tract. In vitro studies have primarily used Caco−2 cells as an intestinal epithelial model [28,29]. However, 2D models, such as Caco−2 cells, do not recapitulate characteristics of native epithelial cells with respect to cytokine production and receptor and transporter expression [30,31,32]. Thus, 3D models, which incorporate stromal cells and extracellular matrix, are becoming widely used as intestinal models, due to the similarity between morphology and behaviour of cells in the 3D model and cells in vivo [32]. Of course, a limitation of this model is that it does not incorporate the gut microbiome and the morphology is not always perfectly resembled, but it is still a more representative model of in vivo effects than 2D cell culture. There are multiple studies on the effects of PM exposure on mice in vivo [28,29,36,49,50], but the composition of the gut microbiome in these animals differs from that of humans, and the intestinal structure of mice differs from that of humans (i.e., longer villi and lack of mucosal folds, etc.). These differences have been nicely reviewed by Nguyen et al. 2015 [34]. Therefore, the purpose of this study was to analyze the effects of PM exposure in 3D human tissues, since these tissues not only have epithelial cells but also other cells that are important for cellular crosstalk such as enterocytes, paneth cell, M cells, tuft cells and intestinal stem cells. 

As reported, we measured the effects of PM exposure on changes in TEER and did not observe differences in exposed tissues compared to unexposed, even after two weeks of exposure (maximum time recommended for culture by manufacturer of 3D EpiIntestinal tissues). These data are in agreement with the findings of Li et al. 2017, since they also did not observe differences in intestinal permeability in vivo in mice after weeks of PM exposure (10 weeks, three times/week) [29]. Importantly, this observation does not mean that PM exposure did not result in morphological or biochemical effects on the exposed tissues. Indeed, it is possible that the morphological changes and loss in barrier functions are evident as a consequence of the biochemical effects that we have observed for ZO-1, claudin 1, and desmocollin. Possibly, extending the time of culture and exposure would have resulted in TEER increase, but as a model limitation it was not possible to extend the experiments for more than two weeks. 

Previous work from our lab has demonstrated that PM exposure can increase levels of the lipid peroxidation marker 4HNE by altering redox homeostasis in other 3D tissue models [37,51]. In the present study, we also observed increased levels of 4HNE in tissues exposed to PM for one week and two weeks. The ability of PM to induce tissue oxidative stress is mainly a consequence of the transition metals present in the surface of the particles. As suggested, the interaction between the particles and the cells could generate oxidative molecules either directly (Fenton reaction) or indirectly via the activation of the AhR receptor. The latter is a ligand activated transcription factor involved in the detoxification of xenobiotics by the activation of monooxygenases enzymes that can produce oxidative molecules during the detoxification process [51].

Interestingly, one of the links that has been hypothesized between pollution exposure and the GI tract is a leaky gut, since intestinal permeability has been connected to oxidative stress [52]. Therefore, we focused on whether PM exposure affected cell-cell connectivity. We assessed whether levels of tight junction proteins ZO−1 and claudin decreased in response to PM exposure and observed downregulation of these proteins. Interestingly, decreased levels of tight junction proteins in the gut can result in chronic inflammation and gastric hyperplasia, potentially leading to cancer development [52]. In addition, we observed decreased levels of the desmosome component desmocollin, in response to PM exposure, which can also contribute to inflammation and gastric cancer [53]. However, the mechanism by which PM exposure reduces protein levels of these adhesion proteins is unclear and merits further research. 

It is possible that these cell–cell adhesion proteins can be post-translationally modified in response to PM exposure. For instance, 4HNE can form adducts with proteins, leading to protein degradation [39]. In addition, due to its capacity to induce signalling cascades [39], increased levels of 4HNE could also result in activation of mitogen-activated protein kinases which can phosphorylate claudin-1 and alter its subcellular localization [54]. Other potential mechanisms directly or indirectly related to PM exposure that could alter localization and/or levels of these proteins include post-translational modifications, such as ubiquitination, sumoylation, and palmitoylation [54]. It is also possible that PM exposure alters messenger levels of these proteins. Alternatively, it has been previously demonstrated that exposure of Caco-2 cells to PM increases ROS generation, increasing activity of the redox-sensitive transcription factor NF-κB [28]. This transcription factor can regulate transcription of myosin light chain kinase (MLCK), resulting in reorganization of proteins at tight junctions and alteration of gut permeability [55,56]. Further research is required to test whether regulation of MLCK is involved in the mechanism of PM-induced downregulation of adhesion proteins. 

## 5. Conclusions

In conclusion, we did not observe differences in transepithelial electrical resistance (TEER) between PM exposed and unexposed tissues, regardless of the length of exposure, likely due to the complexity of our model. Nonetheless, in support of previous work [28,36,37], we observed increased levels of the oxidative stress marker 4−hydroxynonenal (4HNE) adducts in tissues exposed to PM, suggesting that a loss of TEER is not necessary for disruption of redox homeostasis. Consistent with a link between loss of redox homeostasis and impaired barrier function, we observed decreased levels of tight junction proteins zonula occludens protein 1 (ZO−1) and claudin-1 in tissues exposed to PM for one week and two weeks. Furthermore, we observed decreased levels of desmosome-associated protein desmocollin in tissues exposed to PM for one week and two weeks. This research suggests that chronic PM exposure could possibly affect GI tract redox homeostasis and alter the levels of proteins involved in cell−cell adhesion, potentially contributing to a compromised tissue barrier that has been observed in several intestinal conditions such as Crohn’s disease, inflammatory bowel syndrome, and colorectal cancer, all characterized by a “leaky gut” clinical feature.
